# Human Cytomegalovirus (HCMV) induces Human Endogenous Retrovirus (HERV) transcription

**DOI:** 10.1186/1742-4690-10-132

**Published:** 2013-11-12

**Authors:** Alice Assinger, Koon-Chu Yaiw, Ingmar Göttesdorfer, Christine Leib-Mösch, Cecilia Söderberg-Nauclér

**Affiliations:** 1 Center for Molecular Medicine, Department of Medicine, Karolinska Institutet, SE-171 76 Stockholm, Sweden; 2 Institute of Virology, Helmholtz Zentrum München, German Research Center for Environmental Health, Neuherberg, Germany; 3 Department of Hematology and Oncology, University Hospital Mannheim, University of Heidelberg, Mannheim, Germany

**Keywords:** Cytomegalovirus, Retrovirus, Reverse transcriptase, HERV, Cancer, Endothelial cells, Monocytes

## Abstract

**Background:**

Emerging evidence suggests that human cytomegalovirus (HCMV) is highly prevalent in tumours of different origin. This virus is implied to have oncogenic and oncomodulatory functions, through its ability to control host gene expression. Human endogenous retroviruses (HERV) are also frequently active in tumours of different origin, and are supposed to contribute as cofactors to cancer development. Due to the high prevalence of HCMV in several different tumours, and its ability to control host cell gene expression, we sought to define whether HCMV may affect HERV transcription.

**Findings:**

Infection of 3 established cancer cell lines, 2 primary glioblastoma cells, endothelial cells from 3 donors and monocytes from 4 donors with HCMV (strains VR 1814 or TB40/F) induced reverse transcriptase (RT) activity in all cells tested, but the response varied between donors. Both, gammaretrovirus-related class I elements HERV-T, HERV-W, HERV-F and ERV-9, and betaretrovirus-related class II elements HML-2 - 4 and HML-7 - 8, as well as spuma-virus related class III elements of the HERV-L group were up-regulated in response to HCMV infection in GliNS1 cells. Up-regulation of HERV activity was more pronounced in cells harbouring active HCMV infection, but was also induced by UV-inactivated virus. The effect was only slightly affected by ganciclovir treatment and was not controlled by the IE72 or IE86 HCMV genes.

**Conclusions:**

Within this brief report we show that HCMV infection induces HERV transcriptional activity in different cell types.

## Findings

Human cytomegalovirus (HCMV) is a ubiquitous virus infecting 40-100% of the world’s population. It usually causes a mild or asymptomatic infection, but may cause severe and life-threatening disease in immunocompromised hosts [1]. Emerging evidence today implies that HCMV can be detected in very high prevalence in cancers of different origin e.g. glioblastoma, medulloblastoma, neuroblastoma, colon, breast and prostate cancer [2-10]. It is currently debated whether HCMV is oncogenic or oncomodulatory in human cancer, although it fulfills the modified criteria for Koch’s postulates for human tumour viruses [11,12]. Other viruses implied as tumour viruses include Epstein Barr virus (EBV), Hepatitis B and C, human papillomavirus, human herpes virus 8 (HHV-8), Merkel cell polyomavirus and human T-lymphotropic virus type 1 (HTLV-1) and recently human endogenous retroviruses (HERV) [13-15].

HERVs and related retrotransposons constitute approximately 8% of the human genome [16,17]. Most HERVs are defective and generally not considered to be infectious [16,18], but are transmitted vertically. While they are known to be transcriptionally active during embryonic development, they are generally down-regulated in adult human tissues by epigenetic mechanisms such as DNA methylation or chromatin modifications [19,20]. However, induction of HERV transcription is possible under certain circumstances, and may have a possible role in some pathological conditions. For example, an increased prevalence of several HERVs in cancer has led to studies of a potential role of HERVs in tumour development (reviewed in [21,22]) and investigation into the idea that HERVs could be potential therapeutic targets as they represent virus-like tumour antigens. In particular, activation of the HERV-K(HML-2) group has been detected in high prevalence in breast and bladder cancer, sarcoma, malignant melanoma and lymphoma (reviewed in [23]). This HERV group is considered as the most complete and biologically most active. Even though not infectious, some proviruses of this group are able to form retroviral particles, and their gene products could be pathogenic and might play a role in various human cancers [22]. For example HERV-K(HML-2) has been implicated in the initiation of malignant transformation and disease progression in melanoma [24,25].

It is widely unclear how HERV gene expression is controlled or induced in pathological processes, including cancer. Due to the high prevalence of HCMV in different tumours, and the ability of this virus to control host cell gene expression, we sought to define whether HCMV affects HERV expression.

**Figure 1:**
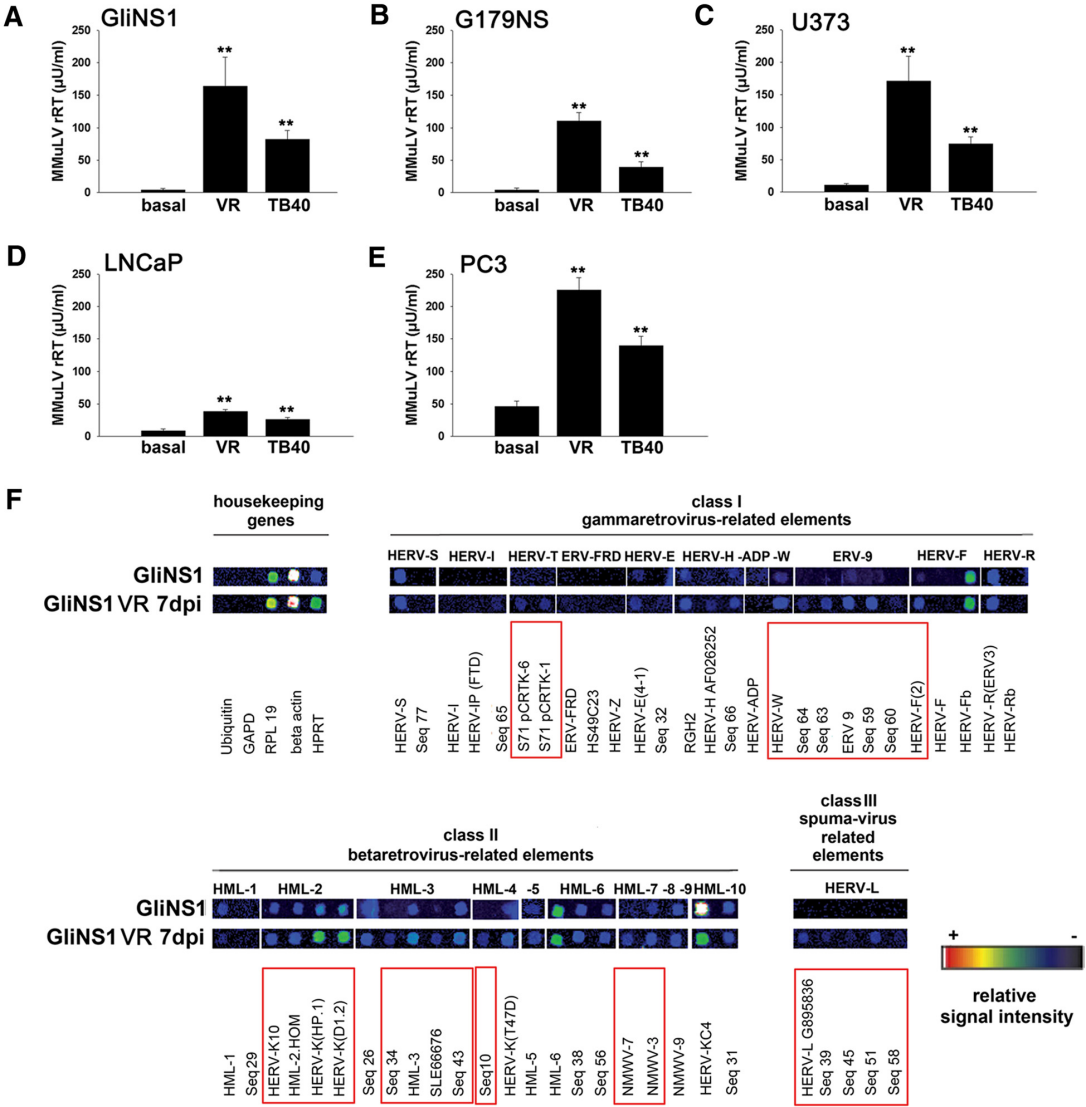
**Effects of HCMV on the activity of Mn**^**2+**^**-dependent RT and HERV transcription profile in human cancer cell lines. A**, **B**: RT activity in glioma initiating cells GliNS1 **(A)** and G179NS **(B)** 5 days post infection with 1 MOI HCMV strain VR 1814 or TB40/F; **D**, **E**: RT activity in prostate cancer cells PC3 **(E)** and LNCaP **(D)**; **C**: RT activity in glioma cell line U373 5 days post infection with 1 MOI HCMV strain VR 1814 or TB40/F; means and standard deviation of 5 independent experiments **F**: One representative example of false-colour microarray data sets representing uninfected and HCMV - infected (VR 1814, MOI 1, 7 dpi) samples. Detailed information about the identity of microarray capture probes has been described previously [26,27] and can be found in the (Additional file 1). Each positive spot on the microarray represents multiple HERV loci assigned to one subgroup of multicopy HERV elements with sufficient sequence homology. The housekeeping genes upiquitin, GAPD, RPL19 and HPRT served as an internal control. Differences between uninfected and infected cells are indicated by red boxes. **p = <0.01.

**Figure 2:**
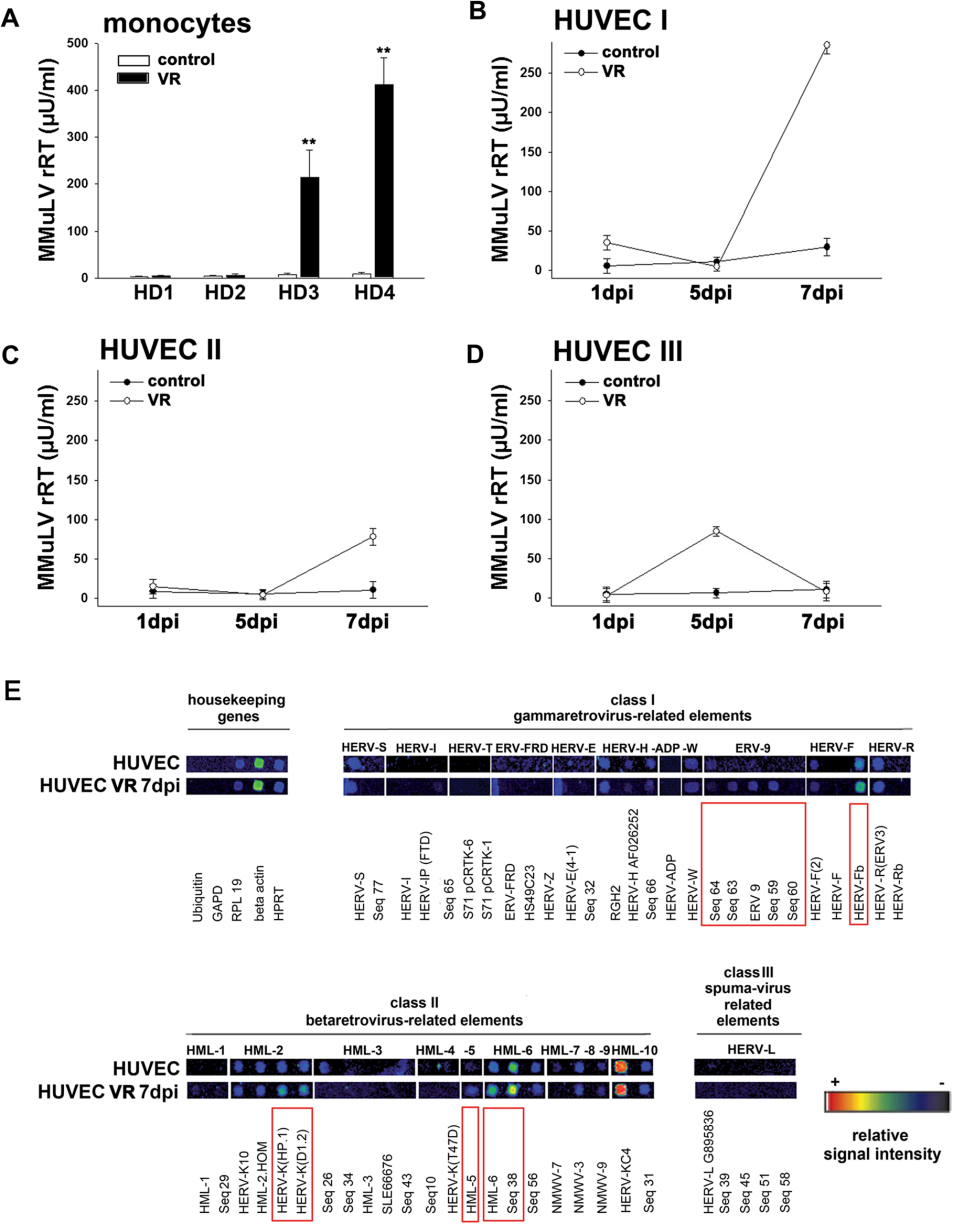
**Effects of HCMV on the activity of Mn**^**2+**^**-dependent RT and HERV transcription profile in cells from healthy donors. A)** RT activity in monocytes from 4 healthy donors (HD1-4) 5 days post infection with 1 MOI HCMV strain VR; **B**-**D**: RT activity in HUVEC from 3 healthy donors (I-III) 1–7 days post infection with 1 MOI HCMV strain VR; means and standard deviation of 2 independent infections in triplicates; **E**: HERV transcription profile of uninfected and HCMV infected HUVECs: One representative example of false-colour microarray data sets representing uninfected and HCMV - infected (VR 1814, MOI 1, 7 dpi) samples. Differences between uninfected and infected cells are indicated by red boxes. For detailed information see Figure 1 and (Additional file 1). **p = <0.01.

We found that HCMV infection by two different clinical HCMV isolates, VR1814 and TB40/F, induced HERV activity in all cancer cell lines tested (for detailed methods see Additional file 1). Reverse transcriptase activity was highly induced by both HCMV strains in neural tumour stem cells, GliNS1 and G179NS, astrocytoma cell line, U373, and the prostate adenocarcinoma cells LNCaP and PC3 (Figure 1A-E). HCMV also induced reverse transcriptase activity in human umbilical vein endothelial cells (HUVEC) and blood-derived monocytes from healthy individuals, with strong individual variations in the levels and kinetics of HERV expression between donors (Figure 2A-D). From these data we conclude that HCMV-induced HERV transcription activity is a general mechanism that can occur in a variety of cell types, although the exact nature of the response varied.

A RetroArray of HCMV-infected neural tumour stem cells (GliNS1) revealed that class I gammavirus-related elements, such as HERV-T, HERV-W, HERV-F and ERV-9 were up-regulated (Figure 1F). Also class II betavirus-related elements such as the HERV-K groups HML-2, -3, -4, -7 and -8 and some class III spuma-virus related HERV-L groups were expressed at higher levels in HCMV-infected versus non-infected GliNS1 cells (Figure 1F). A similar pattern could be observed in HUVEC from healthy donors, where class I HERV groups ERV-9 and HERV-F and class II HERV groups HML-2, -5, and -6 were up-regulated (Figure 2E).

Little is currently known about the induction of HERV-K(HML-2) expression, although CpG methylation status of the HERV promoter or their regulatory elements, have been suggested to be crucial in the regulation of their activity [28,29]. Interestingly, we recently found that HCMV causes a general hypomethylation by regulating DNA methyltransferase (DNMT) 1 and 3 expression in infected cells [30], which may also affect CpG methylation of the HML-2 promoter, or their regulatory elements, and thereby facilitate retroviral activation.

**Figure 3:**
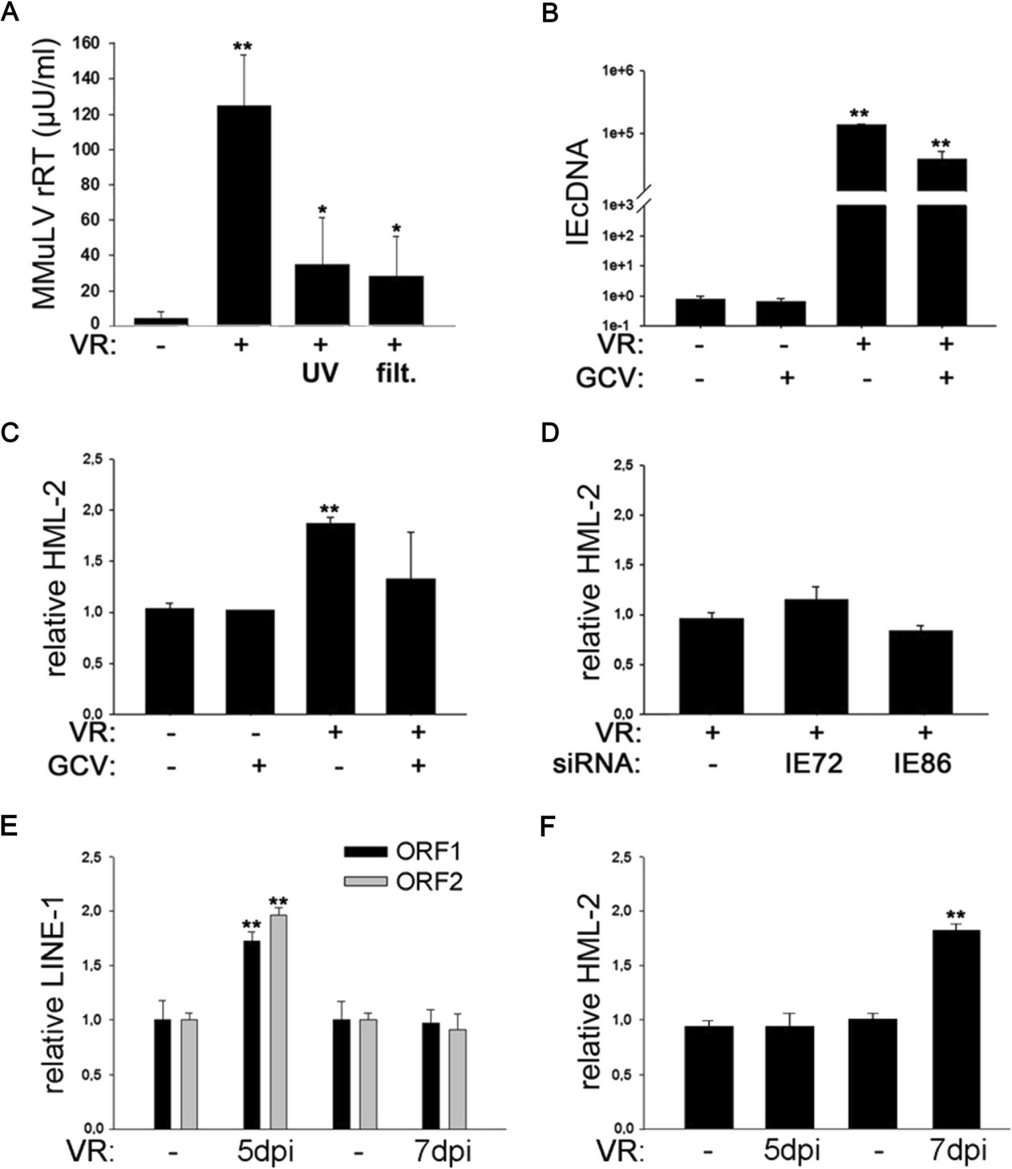
**Effects of HCMV replication and IE on HERV-K(HML-2) transcription. A)** RT activity in glioma initiating cells GliNS1 **(A)** 7 days post infection with 1 MOI HCMV or UV-inactivated HCMV and filtered HCMV (2 μm pore size); (n = 4); **B)** Effect of GCV on IEcDNA expression induced by HCMV strain VR 1814 (n = 3) 7 dpi; **C)** Effect of GCV on HML-2 transcription induced by HCMV strain VR 1814 (n = 3) 7 dpi; **D)** Effects of siRNA-IE72 and siRNA-IE86 on VR 1814 induced HML-2 transcription (n = 2). **E)** Effect of HCMV on LINE-1 expression in GliNS1 at 5 and 7 dpi (n = 3). **F)** Effect of HCMV on HML-2 expression in GliNS1 at 5 and 7 dpi (n = 3). Detailed information on the methods are described in the (Additional file 1). *p = <0.05 **p = <0.01.

To determine if active HCMV replication is necessary to induce HERV expression, we tested the effect of UV-inactivated HCMV and filtered HCMV (0.2 μm pore size) on reverse transcriptase activity. Inactivated virus or filtered HCMV supernatant could partly mimic the effects of HCMV infection on RT activity, but the increase in HERV transcription activity was far less pronounced compared to HCMV infection (Figure 3A).

We further observed a trend for reduced HCMV-induced HERV-K(HML-2) transcription (Figure 3C) when cells were treated with the antiviral agent ganciclovir, implying that early (E) and late (LA) gene products might be involved in HERV expression and RT-activity. Moreover, silencing of immediate early (IE) gene products with siRNA against IE72 and IE86 did not change HCMV-induced HML-2 transcription (Figure 3D), suggesting that IE genes were not involved in controlling HERV transcription in infected cells. Clearly, unidentified soluble molecules induced by HCMV and present in the inoculum, as well as during HCMV infection, could induce HML-2 activity. HCMV is dependent on inflammation for its reactivation, and induces several cytokines and growth factors [31] that may further enhance HERV activation.

In addition to long terminal repeat (LTR)-containing HERVs, Long Interspersed Nuclear Element 1 (LINE-1) may also contribute to the abundant RT activity in human cancers [32]. LINE-1 elements contain two open reading frames (ORFs): ORF1 encodes an RNA binding protein, and ORF2 at least two enzymatic activities, an endonuclease and a reverse transcriptase [33].

To examine whether HCMV not only interferes with HERV expression, but also modulates LINE-1 activity, we determined transcription of LINE-1 ORF1 and ORF2 in response to HCMV infection. While in GliNS1 cells a significant increase of HERV-K(HML-2) transcription was observed 7 days post HCMV infection (Figure 3F), LINE-1 activity peaked at day 5 and was no longer detectable at day 7 post HCMV infection (Figure 3E). These data indicate that HCMV can also modulate LINE-1 expression and suggest that HCMV has an impact on various genomic retroelements. LINE-1 and HERV-K(HML-2) appear to be influenced by HCMV in different ways, and may both play functional but distinct roles in the onset and progression of the tumourigenic process. The lag between LINE-1 and HERV-K(HML-2) activity could point to an interaction between these different types of retrotransposons.

Further studies are required to determine if HCMV causes HERV-K(HML-2) activation in a direct or a LINE-1 dependent fashion and to evaluate whether HCMV or HCMV induced HERVs may play a role in tumour development or progression. HCMV provides oncomodulatory functions, and through numerous immune evasion strategies HCMV may lead to tumour development in cells not undergoing lytic infection. RT inhibiting drugs as well as LINE-1-specific interference have been shown to drastically reduce the tumourigenic potential of melanoma cells in vivo [34].

HERVs are suggested to influence tumour development indirectly e.g. via immunosuppression mediated by the Env proteins [35] or by expression of regulatory proteins such as Rec and Np9 that interact with cellular transcription factors involved in tumourigenesis [22].

A direct effect would depend on horizontal transmission, which has so far only been demonstrated among endogenous animal retroviruses e.g. mouse leukaemia virus, mouse mammary tumour virus and porcine endogenous retroviruses (as reviewed in [15]). Therefore, HERVs are currently mainly considered as possible cofactors in tumour development. Our data suggests that HCMV and an inflammatory environment could facilitate HERV activation in vivo in early tumour lesions.

Regardless of their role in tumourigenesis, their dual presence in certain tumours may present an opportunity for the development of new therapeutic strategies. Valganciclovir treatment in glioblastoma patients improves survival [36], and several immunotherapy trials are ongoing to target HCMV in glioblastoma. Furthermore, similar antigenic determinants are shared between antigen HERV-K-MEL and Bacillus of Calmette Guerin (BCG), vaccinia as well as yellow fever virus. Therefore vaccination against these viruses is implied to mediate a significant, protective effect against HERV positive melanoma (reviewed in [23]). Thus, both HCMV and HERVs may be considered as immunological targets for cancer therapy, while their individual or cooperative role in cancer needs to be further elucidated.

## Competing interests

The authors declare that they have no competing interests.

## Authors’ contributions

AA designed and coordinated the study, performed the RT activity assays, analysed and interpreted the data and wrote the manuscript, KCY established and performed qPCR, ganciclovir and siRNA experiments, analysed and interpreted the data, IG performed the RetroArray and analysed the data, CLM participated in the study design and coordination and helped to analyse and interpret the RetroArray data, CSN conceived the study, participated in the study design and wrote the manuscript. All authors read and approved the final manuscript.

## Supplementary Material

Additional file 1 Materials and methods. Click here for file
