# Supplementary material for: Human Cytomegalovirus (HCMV) induces Human Endogenous Retrovirus (HERV) transcription
Source: Retrovirology. 2013 Nov 12;10:132. doi: 10.1186/1742-4690-10-132 (PMC3842806; doi:10.1186/1742-4690-10-132)
Supplement: Additional file 1 — Materials and methods. [file 1742-4690-10-132-S1.docx]

Additional file 1

**Material and Methods**

**Cell Culture and Virus Propagation**

Human umbilical vein endothelial cells (HUVEC) were isolated cultured as previously described [1], and cultured in Medium 199 supplemented with 20% heat-inactivated fetal bovine serum, 1µg/ml hydrocortisone, 1ng/ml EGF, 100 IU/ml penicillin G, and 100 μg/ml streptomycin (Life Technologies).

Monocytes were isolated from ethylenediaminetetraacetic acid (EDTA) blood, obtained from healthy donors, by gradient density centrifugation with Histopaque 1077 and Histopaque 1119 (both Sigma Aldrich). Peripheral blood mononuclear cells were then seeded onto petri dishes and after 2 hours non-adherent cells were washed away. Adherent monocytes were cultured in AIM-V/IMDM/heat-inactivated AB-serum (6:3:1).

Human prostate cells, PC-3 and LNCaP cells were grown in RPMI medium. Astrocytoma cells, U373 were cultured in DMEM/F-12 medium. Both media were supplemented with 10% heat-inactivated fetal bovine serum, 2 mM l-glutamine, 100 IU/ml penicillin G, and 100 μg/ml streptomycin (Life Technologies). Cells were grown to approximately 50%–60% confluence and infected in low-serum medium for the indicated time with HCMV clinical isolate–like strain VR 1814 or TB40/F at an MOI of 1. Controls were either not exposed to virus or were exposed to UV-inactivated virus (UV Stratalinker 1800; Stratagene) or filtered virus (0.1µM).

HCMV VR 1814 (provided by G. Gerna, University of Pavia, Pavia, Italy) was prepared from supernatants of cultures of in vitro HUVECs, grown in EGM2 medium supplemented with EGM-2 kit (Clonetics, Cambrex). HCMV TB40/F was prepared from supernatants of cultures of in vitro infected MRC-5 cells. Virus containing supernatants were clarified by centrifugation (3 000g, 5min), frozen, and stored at –85°C until use. Viral titres were determined by viral plaque assay [2].

**Reverse transcriptase activity**

Reverse transcriptase activity was determined in the supernatants of different cancer cells as well as HUVECs and monocytes from healthy individuals. Cells were infected with 1 MOI VR 1814 or TB40E and supernatants harvested at the indicated time points. Supernatants were centrifuged (3000g, 5min) and either analysed immediately or stored at -85°C. Reverse transcriptase activity was determined by an HS-Mn RT Activity Kit (Cavidi, Uppsala, Sweden) according to the instructions of the manufacturer. The RT-assay determines all RT activity in a sample, independent of their genomic or non-genomic origin. The assay is based on the quantification of RT-activity by its potential to generate double stranded nucleotide strand from a single RNA strand.

**Retrovirus-specific microarray (RetroArray)**

The Retroarray analysis was performed with uninfected and HCMV-infected (VR1814, 7dpi, MOI 1) GliNS1 or HUVECs from healthy donors. RNA was isolated by RNeasy Mini Kit (Qiagen) according to the instructions of the manufacturer. To rule out any contamination with DNA, lysates were treated with with 100 units/µg RNase-free DNase using the RQ1 RNase-Free DNase Kit (Promega, Mannheim, Germany) and RNA checked for negativity of DNA contamination using primers specific for HERV-L LTR sequences (forward primer: ATCTGGGTGGGCACCATCAAGTTA, reverse primer: TCTCACAATAGGCCATCTGCAAGC). Only DNA-negative RNA samples (1–2 µg) were subsequently transcribed into cDNA using SuperScript III First-Strand Synthesis according to the manufacturer's instructions (Invitrogen, Karlsruhe, Germany).

Hybridisation probe synthesis and labelling by MOP-PCR, as well as DNA chip preparation, hybridisation and post-processing of retrovirus-specific microarrays were performed as described previously [3]. Hybridised microarrays were scanned using an Affymetrix Scanner GMS 418 (laser power 100%, gain 50–60%) and false colour mapping was used for image visualisation.

**Inhibition of HCMV replication and gene products**

GliNS-1 cells were treated with ganciclovir (Hoffmann La Roche Ltd, Basel Switzerland) at 2.5 mM. For IE72 and IE86 knockdown, GliNS1 cells were transfected siRNA (Dharmacon, Lafayette, CO) for 24h followed by infection with HCMV and harvested at 1 dpi.

HCMV IE1-72 and IE2-86 were knocked down in infected GliNS-1 cells by transfection with 2 μl of Lipofectamine 2000 and of 2 μl of 20 μM by small interfering RNAs (siRNAs) over night. IE1-72 siRNA 5′GGUUAUCAGUGUAAUGAAGdTdT-3′ and complement 5′CUUCAUUACACUGAUAACCdTdT-3′ was used to silence HCMV IE1-72 and IE2-86 siRNA oligonucleotide 5′-AAACGCAUCUCCGAGUUGGACdTdT-3′ and complement 5′-GUCCAACUCGAGAUGCGUUUdTdT-3′ targeting for HCMV IE2-86 (all from Dharmacon, Lafayette, CO). At 24 h posttransfection cells were infected with HCMV at MOI 1.

**Quantification of HERV-K(HML-2) and LINE-1 ORF1 and ORF2 transcripts by relative qRT-PCR**

To confirm and extend the results from the RetroArray we also determined HERV-K(HML-2) and LINE-1 ORF1 and ORF2 transcripts by relative qRT-PCR.

QRT-PCR for HML-2 *gag* transcription was performed with the 7900HT Fast Real-Time PCR system (Applied Biosystems), using ABI Fast SYBR Green Mastermix. A 30 sec initial denaturation step at 95°C was followed by 40 amplification cycles of 3 sec at 95°C, 20 sec at 60°C. Melting curves were generated for the final PCR products by decreasing the temperature to 60°C for 15 s followed by an increase in temperature to 95°C using forward primer: GGCCATCAGAGTCTAAACCACG and reverse primer: CTGACTTTCTGGGGGTGGCCG as described previously [4]. Fluorescence was measured at auto increments. RNA-Polymerase II (RPII) transcripts were analyzed as internal control (forward primer: gcaccacgtccaatgacat and reverse primer: gtgcggctgcttccataa). ΔCT-values were calculated as follows: CT(RPII)-CT(HERV element), and were normalized to RPII levels.

LINE-1 ORF1 and ORF2 transcription was determined by real-time PCR using TaqMan Fast Universal PCR Master Mix with Applied Biosystems TaqMan MGB (minor groove binder) primers/probe of LINE-1 ORF1 gene (forward primer: TTGGAAAACACTCTGCAGGATATTAT; reverse primer: TTGGCCTGCCTTGCTAGATT; probe: FAM-CAGGAGAACTTCCC-MGB) and LINE-1 ORF2 gene (forward primer: AAAATACTGGCAAACCGAATCC; reverse primer: TGAAGCCCACTTGATCATGGT and probe: FAM-AGCACATCAAAAAGCTTAT-MGB) as described in [5]. Human β2-microglobulin (B2M, assay ID, Hs00984230_m1) was used for normalization. Amplifications were performed starting with a 20 second template denaturation step at 95°C, followed by 40 cycles at 95°C for 1 sec and 60°C for 20 sec in a 7900HT Fast Real-Time PCR system (Applied Biosystems). The results were analyzed with SDS 2.4 software. ΔCT were calculated as follows: CT(LINE-1 ORF1 or ORF2) – CT(B2M). The x-fold induction of HERV and LINE-1 transcription in treated cells was calculated by the 2^-ΔΔCT^ method, with values normalized to RPII or B2M and relative to transcription in untreated cells. Quantitative RT-PCR experiments for each gene were performed at least in triplicate.

**References**

1. Butler LM, Jeffery HC, Wheat RL, Rae PC, Townsend K, Alkharsah KR, Schulz TF, Nash GB, Blackbourn DJ: **Kaposi's sarcoma-associated herpesvirus infection of endothelial cells inhibits neutrophil recruitment through an interleukin-6-dependent mechanism: a new paradigm for viral immune evasion.** *J Virol* 2011, **85:**7321-7332.

2. Wentworth BB, French L: **Plaque assay of cytomegalovirus strains of human origin.** *Proc Soc Exp Biol Med* 1970, **135:**253-258.

3. Seifarth W, Frank O, Zeilfelder U, Spiess B, Greenwood AD, Hehlmann R, Leib-Mosch C: **Comprehensive analysis of human endogenous retrovirus transcriptional activity in human tissues with a retrovirus-specific microarray.** *J Virol* 2005, **79:**341-352.

4. Diem O, Schaffner M, Seifarth W, Leib-Mosch C: **Influence of antipsychotic drugs on human endogenous retrovirus (HERV) transcription in brain cells.** *PLoS One* 2012, **7:**e30054.

5. Carlini F, Ridolfi B, Molinari A, Parisi C, Bozzuto G, Toccacieli L, Formisano G, De OD, Paradisi S, Grober OM et al.: **The reverse transcription inhibitor abacavir shows anticancer activity in prostate cancer cell lines.** *PLoS One* 2010, **5:**e14221.
